# Characteristics and impact of the most-cited palliative oncology studies from 1995 to 2016

**DOI:** 10.1186/s12885-018-5150-7

**Published:** 2018-12-22

**Authors:** Corbin Eule, Nizar Bhulani, Elizabeth Paulk, Ramona Rhodes, Muhammad Shaalan Beg

**Affiliations:** 1 Department of Internal Medicine, UT Southwestern Medical Center, 5323 Harry Hines Boulevard, Dallas, TX 75390 USA; 2 Harold C. Simmons Comprehensive Cancer Center, UT Southwestern Medical Center, 2201 Inwood Road, Dallas, 75390 TX USA

**Keywords:** Palliative oncology, Palliative medicine, Oncology, Research trends

## Abstract

**Background:**

Palliative care, as a relatively young field within medicine, has increasingly used original research to validate and standardize its practice. In particular, palliative care has been incorporated into oncology to better address end-of-life decisions and care. The goal of this study is to identify seminal studies in the field of palliative oncology while more broadly characterizing the trends across the literature.

**Methods:**

The publication databases Scopus and Web of Science were queried using predefined search terms to identify palliative oncology studies published between 1995 and 2016. The 100 most-cited articles from the time periods 1995–2005 and 2006–2016 were selected and analysed for publication data and study content.

**Results:**

Palliative oncology studies were found to primarily examine patients with multiple rather than single cancer types and rarely were randomized controlled trials. Early research topics of pain, symptoms, and survival studies have been replaced by the issues of access to care, healthcare utilization, and religion and spirituality.

**Conclusions:**

By identifying and analyzing notable studies in palliative oncology, we found areas of research that are commonly investigated or overlooked and identified model studies that highlight the need for additional disease-specific randomized control trials to provide high quality clinical evidence in the field.

**Electronic supplementary material:**

The online version of this article (10.1186/s12885-018-5150-7) contains supplementary material, which is available to authorized users.

## Background

The concept of palliative care was notably and quite recently articulated by Dr. Cicely Saunders in her 1960 publication on the subject of caring for dying patients. The resulting chapter “The Management of Patients in the Terminal Stage” was a unique entry in an expansive six-volume series on cancer in the UK. At the time, care for dying patients was limited to a small number of religious hospice organizations, one of which, St. Joseph’s Hospice in Hackney, London, employed Saunders as a research fellow. [1] The term “palliative care” was not adopted until 1974, when Dr. Balfour Mount from The Royal Victoria Hospital of McGill University in Montreal, Canada, popularized it in order to avoid the perceived connotations of the word hospice. This burgeoning field gained heightened attention 10 years later when the Institute of Medicine published the report “Approaching Death: improving care at the end of life” (M.I. Field and C.K. Cassel, editors), which documented deficiencies in end-of-life care in the United States. Despite this relatively short history as a distinct and officially recognized field within medicine, palliative care is rapidly and broadly being incorporated into the practice models of medical institutions worldwide. Only in 2006 was Hospice and Palliative Medicine recognized as an official subspecialty by the American Board of Medical Specialties. [2] Today, palliative care has increasingly become a focus of investigation as reflected by the growth in medical literature published in the field. [3]

Characterizing the direction of research and advances in palliative care presents a uniquely challenging task. [4] Palliative care, as defined by the World Health Organization, “is an approach that improves the quality of life of patients and their families facing the problem associated with life-threatening illness, through the prevention and relief of suffering by means of early identification and impeccable assessment and treatment of pain and other problems, physical, psychosocial and spiritual.” [5] Given its broad implications for all fields of medicine, palliative care is often wide-ranging in its scope and publication. In order to identify the most salient information, certain specialties have analysed how palliative care is specifically being employed or investigated within their field. [6] Oncology, in particular, is often associated with end-of-life decisions and care, and therefore, has great utility for palliative care in the management of the symptoms and stressors of life-limiting illness. A central tenant of conventional oncology care is focused on improving quality of life and survival by modifying the natural course of cancer through cancer directed treatments: systemic therapy, radiation, and surgery. The established framework of medical, surgical, and radiation oncology in cancer care may have led to a delay in integration of distinct palliative care services into the field of oncology.

Previous reports on palliative care in oncology have not comprehensively evaluated studies, identified seminal works, and trended their impact in the field. [7] In this paper we define and map the evolving trends within the palliative oncology literature. In order to achieve this goal, we have identified and characterized the 100 most-cited palliative oncology studies published between 1995–2005 and 2006–2016.

## Methods

The search terms (palliat$ OR hospice$ OR “terminal care”) developed by Sladek et al. were used to filter publications specific to palliative care from the general medical literature. [8] This expression was combined via “AND” with the terms (adenocarcinoma* OR adenosarcoma* OR antineoplastic OR cancer* OR carcinoid* OR carcinoma* OR glioblastoma* OR *cytoma OR glioma* OR hodgkin* OR leukaemi* OR leukemi* OR lymphoma* OR malignan* OR melanoma* OR metasta* OR myelodysplas* OR myeloma* OR neoplas* OR non-hodgkin* OR nonhodgkin* OR nonseminoma* OR oncolog* OR osteosarcoma* OR sarcoma* OR seminoma* OR teratoma* OR tumor* OR tumour* OR waldenstrom* OR mesothelioma* OR blastoma* OR wilms*) to select for palliative medicine literature specifically applied to the field of oncology.

On October 9, 2017, this Boolean expression was used to search the Scopus (http://www.scopus.com) database with the Article title, Abstract, Keywords input. The results were filtered by publication date in the timeframe 1995–2005 and subsequently sorted by Cited by (highest). Each result was reviewed in descending order until 100 articles had been identified that met the study’s inclusion criteria. An article had to present original research in palliative medicine as it relates to oncology. Systematic reviews, case studies, review articles, and opinion pieces were excluded from the study. Studies addressing treatment (e.g. palliative chemotherapy) or other measures specifically intended to be life-prolonging were excluded as well. Only English-language studies were included. The same process was repeated for the years 2006–2016.

The aforementioned Boolean expression was also used to perform a Topic search in the database Web of Science (http://www.webofscience.com). Results were again divided into the two timeframes, sorted by Times Cited – highest to lowest, and selected based on the study inclusion criteria. The Web of Science results were cross-referenced with the Scopus output. Duplicates were excluded, and after inclusion criteria was met, articles were selected based on number of citations until a list of the 100 most-cited palliative oncology articles for each timeframe was compiled.

For each article, the citation count, author names, journal of publication, affiliated institution, country, and year were extracted using the information available from Scopus, Web of Science, and PubMed. Population data for each country of publication was retrieved from the United Nations *World Population Prospects*. [9, 10] In order to further characterize each study, the cancer type, study topic, study population, study design, and data collection were categorized based on a review of the article’s abstract. If further clarification was required, the study manuscript was reviewed in its entirety. Data was analyzed using *Microsoft Excel 2017*.

## Results

The median number of citations from the 100 articles published between 1995 and 2005 was 142 with a range of 91 to 687. Of those, 18 articles were cited more than 250 times, and 6 were cited more than 500 times. From 2006 to 2016, the median number of citations was 78 with a range of 53 to 2453. Only 6 articles were cited more than 250 times, 2 of which were cited over 500 times. The vast majority (*n* = 66) were cited less than 100 times in this time frame (Additional files 1 and 2). The 10 most-cited studies in 1995–2005 and 2005–2016 were identified as potential seminal works in the field of palliative oncology (Tables 1 and 2). The sample studies from 1995 to 2016 were compared as a whole and ranked based on number of citations per year (Table 3).

**Table 1:** Top 10 most-cited palliative oncology studies in 1995–2005

Rank	Citations	First Author	Senior Author	Title	Year	Journal	Institution	Country
1	687	Wolfe, J.	Weeks, J.C.	Symptoms and suffering at the end of life in children with cancer	2000	New England Journal of Medicine	Dana-Farber Cancer Institute	United States
2	675	Bernabei, R.	Mor, V.	Management of pain in elderly patients with cancer	1998	Journal of the American Medical Association	Università Cattolica del Sacro Cuore	Italy
3	635	Christakis, N.A.	Lamont, E.B.	Extent and determinants of error in doctors’ prognoses in terminally ill patients: prospective cohort study	2000	British Medical Journal	University of Chicago	United States
4	580	Breitbart, W.	Brescia, R.	Depression, hopelessness, and desire for hastened death in terminally ill patients with cancer	2000	Journal of the American Medical Association	Memorial Sloan-Kettering Cancer Center	United States
5	528	Lunney, J.R.	Guralnik, J.M.	Patterns of functional decline at the end of life	2003	Journal of the American Medical Association	National Institutes of Health	United States
6	516	Earle, C.C.	Weeks, J.C.	Trends in the aggressiveness of cancer care near the end of life	2004	Journal of Clinical Oncology	Dana-Farber Cancer Institute	United States
7	370	Maltoni, M.	Kaasa, S.	Prognostic factors in advanced cancer patients: Evidence-based clinical recommendations - A study by the steering committee of the European Association for Palliative Care	2005	Journal of Clinical Oncology	Morgagni-Pierantoni Hospital	Italy
8	346	Vainio, A.	Auvinen, A.	Prevalence of symptoms among patients with advanced cancer: An International Collaborative Study	1996	Journal of Pain and Symptom Management	World Health Organization	Switzerland
9	341	de Stoutz, N.D.	Suarez-Almazor, M.	Opioid rotation for toxicity reduction in terminal cancer patients	1995	Journal of Pain and Symptom Management	Medizinische Klinik C, Kantonsspital	Switzerland
10	338	Murray, S.A.	Clausen, H.	Dying of lung cancer or cardiac failure: Prospective qualitative interview study of patients and their carers in the community	2002	British Medical Journal	University of Edinburgh	United Kingdom

**Table 2:** Top 10 most-cited palliative oncology studies in 2006–2016

Rank	Citations	First Author	Senior Author	Title	Year	Journal	Institution	Country
1	2453	Temel, J.S.	Lynch, T.J.	Early palliative care for patients with metastatic non-small-cell lung cancer	2010	New England Journal of Medicine	Massachusetts General Hospital	United States
2	946	Wright, A.A.	Prigerson, H.G.	Associations between end-of-life discussions, patient mental health, medical care near death, and caregiver bereavement adjustment	2008	Journal of the American Medical Association	Dana-Farber Cancer Institute	United States
3	387	Balboni, T.A.	Prigerson, H.G.	Religiousness and spiritual support among advanced cancer patients and associations with end-of-life treatment preferences and quality of life	2007	Journal of Clinical Oncology	Dana-Farber Cancer Institute	United States
4	382	Zhang, B.	Prigerson, H.G.	Health care costs in the last week of life associations with end-of-life conversations	2009	Archives of Internal Medicine	Dana-Farber Cancer Institute	United States
5	301	Heyland, D.K.	Lam, M.	What matters most in end-of-life care: Perceptions of seriously ill patients and their family members	2006	Canadian Medical Association Journal	Kingston General Hospital	Canada
6	262	Wright, A.A.	Prigerson, H.G.	Place of death: Correlations with quality of life of patients with cancer and predictors of bereaved caregivers’ mental health	2010	Journal of Clinical Oncology	Dana-Farber Cancer Institute	United States
7	247	Connor, S.R.	Iwasaki, K.	Comparing Hospice and Nonhospice Patient Survival Among Patients Who Die Within a Three-Year Window	2007	Journal of Pain and Symptom Management	National Hospice and Palliative Care Organization	United States
8	233	Mack, J.W.	Prigerson, H.G.	End-of-life discussions, goal attainment, and distress at the end of life: Predictors and outcomes of receipt of care consistent with preferences	2010	Journal of Clinical Oncology	Dana-Farber Cancer Institute	United States
9	228	Temel, J.S.	Pirl, W.F.	Longitudinal perceptions of prognosis and goals of therapy in patients with metastatic non-small-cell lung cancer: Results of a randomized study of early palliative care	2011	Journal of Clinical Oncology	Massachusetts General Hospital Cancer Center	United States
10	226	Braun, M.	Rodin, G.	Hidden morbidity in cancer: Spouse caregivers	2007	Journal of Clinical Oncology	Hadassah University Hospital	Israel

**Table 3:** Top 10 palliative oncology studies published between 1995 and 2016 as determined by number of citations per year

Rank	Citations/Year	First Author	Senior Author	Title	Year	Journal	Institution	Country
1	362.1	Temel, J.S.	Lynch, T.J.	Early palliative care for patients with metastatic non-small-cell lung cancer	2010	New England Journal of Medicine	Massachusetts General Hospital	United States
2	107.8	Wright, A.A.	Prigerson, H.G.	Associations between end-of-life discussions, patient mental health, medical care near death, and caregiver bereavement adjustment	2008	Journal of the American Medical Association	Dana-Farber Cancer Institute	United States
3^a^	80.6	Bakitas, M.A.	Ahles, T.A.	Early Versus Delayed Initiation of Concurrent Palliative Oncology Care: Patient Outcomes in the ENABLE III Randomized Controlled Trial	2015	Journal of Clinical Oncology	University of Alabama- Birmingham	United States
4	49.1	Zhang, B.	Prigerson, H.G.	Health care costs in the last week of life associations with end-of-life conversations	2009	Archives of Internal Medicine	Dana-Farber Cancer Institute	United States
5	41.0	Wolfe, J.	Weeks, J.C.	Symptoms and suffering at the end of life in children with cancer	2000	New England Journal of Medicine	Dana-Farber Cancer Institute	United States
6^a^	40.4	Greer, J.A.	Temel, J.S.	Effect of early palliative care on chemotherapy use and end-of-life care in patients with metastatic non-small-cell lung cancer	2012	Journal of Clinical Oncology	Massachusetts General Hospital	United States
6	40.4	Earle, C.C.	Weeks, J.C.	Trends in the aggressiveness of cancer care near the end of life	2004	Journal of Clinical Oncology	Dana-Farber Cancer Institute	United States
8	39.6	Balboni T.A.	Prigerson H.G.	Religiousness and spiritual support among advanced cancer patients and associations with end-of-life treatment preferences and quality of life	2007	Journal of Clinical Oncology	Dana-Farber Cancer Institute	United States
9	39.5	Temel, J.S.	Pirl, W.F.	Longitudinal perceptions of prognosis and goals of therapy in patients with metastatic non-small-cell lung cancer: Results of a randomized study of early palliative care	2011	Journal of Clinical Oncology	Massachusetts General Hospital Cancer Center	United States
10	38.7	Wright, A.A.	Prigerson, H.G.	Place of death: Correlations with quality of life of patients with cancer and predictors of bereaved caregivers’ mental health	2010	Journal of Clinical Oncology	Dana-Farber Cancer Institute	United States

^a^Did not appear in top 10 most-cited palliative oncology studies of 2006–2016

Between 1995–2005 and 2006–2016 (Table 4), studies predominantly used mixed cancer types (3 or more) to answer research questions, while lung cancer was most likely to be addressed independently. The subjects of pain and symptoms decreased between the two time frames, but the topic of survival decreased the most both in absolute and relative terms. There has been an increase in studies addressing access to palliative care, end-of-life discussions, religion and spirituality, and healthcare utilization. The psychosocial experience of cancer and palliative care remained a popular subject across both time frames. New study populations of hospitals and countries were examined in 2006–2016, but geriatric patients were less represented as compared to 1995–2005. In regard to study design, there was a decrease in prospective cohorts nearly equivalent to an increase in randomized control trials. Clinical means of data collection, such as physician assessment or laboratory and exam data, were used less frequently, while pre-existing datasets obtained via chart review or national and local databases were increasingly utilized.

**Table 4:** Characteristics of the most-cited palliative oncology studies published in 1995–2005 and 2006–2016

	1995–2005	2006–2016	Relative Change	Absolute Change
Cancer Type				
brain	0	4		4
colon	3	2	0.67	−1
lung	9	10	1.11	1
mixed	89	86	0.97	−3
prostate	1	0		−1
Study Topic				
access to care	6	15	2.50	9
cost	1	3	3.00	2
delirium	3	1	0.33	−2
dementia	1	0	0.00	−1
end-of-life discussions	4	9	2.25	5
physician-assisted death	2	0	0.00	−2
frailty	2	1	0.50	−1
integrative medicine	1	2	2.00	1
pain	10	6	0.60	−4
place of death	9	8	0.89	−1
prognosis	14	3	0.21	−11
psychiatric symptoms	8	9	1.13	1
psychosocial	19	23	1.21	4
quality of life	9	10	1.11	1
racial/ethnic disparities	3	3	1.00	0
religion/spirituality	1	11	11.00	10
symptoms	20	12	0.60	−8
healthcare utilization	4	15	3.75	11
Study Population				
adult patients	67	75	1.12	8
caregivers	22	19	0.86	−3
countries	0	1		1
geriatric patients	6	2	0.33	−4
hospitals	0	4		4
pediatric patients	3	4	1.33	1
physicians	13	11	0.85	−2
nurses	2	2	1.00	0
Study Design				
cross-sectional	35	34	0.97	−1
prospective cohort	38	28	0.74	−10
randomized control trial	7	16	2.29	9
retrospective cohort	20	22	1.10	2
Data Collection Method				
chart review	5	11	2.20	6
clinical	32	23	0.72	−9
database	11	16	1.45	5
interview	24	25	1.04	1
survey	31	34	1.10	3

The majority of palliative oncology research is concentrated in the United States (Tables 5 and 6), particularly in the Harvard-affiliated Boston medical institutions (Table 7). The United Kingdom, Canada, and Italy together with United States yielded the majority of publications in the field in both 1995–2005 and 2006–2016 (77% and 74%, respectively). However, the most studies per total country population were published in Switzerland in 1995–2005 and Canada in 2006–2016. The 5 most published authors within the selected studies were a distinct set of researchers and did not reappear in either time frame (Table 8). Over both periods, the *Journal of Clinical Oncology* (*n* = 40) and the *Journal of Pain and Symptom Management* (*n* = 29) were the most frequent sources of publication (Table 9).

**Table 5:** Palliative oncology study publication by country and country population in 1995–2005

Country	Publications	Population in Millions [9]	Publications per Million Population
United States	48	298.213	0.16
United Kingdom	16	59.668	0.27
Canada	12	32.268	0.37
Italy	10	58.093	0.17
Australia	6	20.155	0.30
Japan	5	128.085	0.04
Sweden	3	9.041	0.33
Switzerland	3	7.252	0.41
Spain	2	43.064	0.05
Taiwan	2	*unavailable*	
Finland	1	5.249	0.19
Israel	1	6.725	0.15
Netherlands	1	16.299	0.06
Norway	1	4.620	0.22

**Table 6:** Palliative oncology study publication by country and country population in 2006–2016

Country	Publications	Population in Millions [10]	Publications per Million Population
United States	59	321.774	0.18
United Kingdom	12	64.716	0.19
Canada	10	36.940	0.27
Italy	6	59.798	0.10
Japan	4	126.573	0.03
Netherlands	4	16.925	0.24
Australia	4	23.969	0.17
South Korea	2	50.293	0.04
Spain	3	46.122	0.07
Sweden	2	9.779	0.20
Taiwan	3	23.381	0.13
Belgium	2	11.299	0.18
Germany	2	80.689	0.02
Israel	2	8.064	0.25
Denmark	1	5.669	0.18
Switzerland	1	8.299	0.12

**Table 7:** Number of most-cited palliative oncology studies published from top 5 affiliated institutions in 1995–2005 and 2006–2016

1995–2005		2006–2016	
Dana-Farber Cancer Institute	6	Harvard Medical School	17
Harvard Medical School	5	Dana-Farber Cancer Institute	17
Brigham and Women’s Hospital	5	Brigham and Women’s Hospital	10
University of Alberta	5	University of Texas M.D. Anderson	6
Serei Mikatabara Hospital	4	University of Texas Southwestern Medical Center	6
University of Texas M.D. Anderson	4	Massachusetts General Hospital	6

**Table 8:** Top 5 most published authors of the most-cited palliative oncology studies in 1995–2005 and 2006–2016

1995–2005		2006–2016	
Bruera, E.	9	Block, S.D.	13
Weeks, J. C.	7	Prigerson, H.G.	12
Emanuel, E. J.	6	Wright, A.A.	11
Maltoni, M.	5	Balboni, T.A	10
Addington-Hall, J.	5	Paulk, M.E.	8

**Table 9:** Top 5 most frequent sources of publication for the most-cited palliative oncology studies in 1995–2005 and 2006–2016

1995–2005		2006–2016	
Journal of Pain and Symptom Management	19	Journal of Clinical Oncology	28
Palliative Medicine	13	Journal of Pain and Symptom Management	10
Journal of Clinical Oncology	12	Cancer	6
Cancer	7	Journal of Palliative Medicine	5
Journal of the American Medical Association	6	Journal of the American Medical Association	4

## Discussion

In our analysis of palliative oncology literature, the studies identified in our search were subdivided based on publication date, either from 1995–2005 or 2006–2016. The 100 most-cited articles from each time frame were selected in order to limit the scope to the most impactful studies in the field. Each study was systemically examined and categorized with the intent of characterizing palliative oncology literature collectively, charting trends within the field, and elucidating new avenues for subsequent research. In such a nascent field, early studies have primarily sought to substantiate the benefits of palliative oncology care. As a result, this foundational work has contributed to an increased awareness of palliative oncology research and its application to patient care.

Across all publications included in our study, 8 articles were cited over 500 times. As expected, these predominantly occur in the 1995–2005 timeframe, which would allow each study to accumulate more citations in a time dependent manner. All but 1 of the articles were published after 1999, and the 2 overall most-cited articles by Temel J.S. et al. and Wright A.A. et al. were published in 2010 and 2008, respectively. This suggests an increased interest in palliative oncology in scientific investigation over the past decade as indicated both by their number of citations and citation rates.

In both time frames, 1995–2005 and 2006–2016, the analysed studies primarily used mixed cancer types in their inclusion criteria. Not selecting for specific cancer types facilitates a large enough patient population to generate a sufficiently powered study. This pragmatic approach to patient recruitment may reflect the general increase in clinical oncology research. Research protocols which limit participation in additional studies or trial fatigue on the part of patients may impact the type and quantity of patients available for study. Nonetheless, the underlying premise appears to be that type of cancer would have limited influence on the administration of palliative care as opposed to having cancer in general. Lung cancer, in contrast, may have been the most frequently used specific cancer type because its experiences and symptoms present a unique scenario for receiving palliative care. Although a more intuitive explanation for studying palliative care in lung cancer is simply because more patients are available for study due to overall cancer epidemiology and prevalence. [11] If principles of palliative care are held to be largely universal in their application to oncology, the mixed cancer approach is appropriate. Nonetheless, cancer, more so than many other medical problems, represents a very heterogeneous set of diseases, each with a very distinct set of palliative needs. This stems from differences in comorbidity, life style, cancer symptoms, and treatment complications, which vary widely from one cancer to the next. Therefore, future investigation of palliative care in oncology should focus on specific cancer types in order to better delineate the impact of treatment.

After classifying the topics of each study, several trends were suggested from the outcome of this analysis. Studies performed in the later time period were less likely to study the role of prognosis in palliative care. This decrease may correspond with the publication of the seminal paper by Christakis at el. in 2000 which found that only 20% of physicians demonstrated accurate prognostic capability at the end of life. Based on the conclusions of this study, many investigators may have forgone attempts at additional prognostic models and have focused their efforts on other areas. Alternatively, the benefit of early integration of palliative care in advanced disease management may be increasingly accepted, decreasing the utility of prognosis for this purpose. Pain and symptom management had numerous studies between 1995–2005. Research in this area may have waned due to a dearth of new drug development and the challenge of studying subjective criteria such as pain and symptoms, which are particularly susceptible to the placebo effect. Then in 2006–2016, an uptick in studies about religion and spirituality occurred. Although there does not appear to be a clear antecedent in the literature, there is clearly a growing awareness of and interest in understanding the role of spirituality in the mental health and physical experience of patients and families living with serious illness. The work of prolific authors, such as Prigerson H.G., on this topic may also have a disproportionate effect on shifting the body of research in the relatively small field of palliative care.

A particularly interesting topic is the example of end-of-life discussions. This exact phrase did not appear in any of the studies from 1995–2005 but was referred to in more general terms such as “communication.” This heightened emphasis and validation of end-of-life discussions contributed to the inclusion of advance care planning for the first time on the Medicare physician fee schedule in 2016. [12] The increasing acceptance of the proven benefits of palliative oncology likely contributed to the increase in studies assessing the effectiveness or limitations in providing access to its services. The topic of health care utilization was increasingly studied more recently. Emergency department visits, hospitalization, admission to the intensive care unit, and other metrics of care were often used a proxy for cost and aggressive medical intervention for patients at the end of life. Lastly, the psychosocial aspects of palliative oncology remained the consistent and most frequent study topic. These studies often reflected on the holistic experience of cancer and the ability of palliative care to alleviate the dying process. Overall, the distribution of study topics increased between 1995–2005 and 2006–2016, indicating a diversification of the research being undertaken in this field.

In order to address the aforementioned topics, investigators did not limit their studies exclusively to patients (although this represented the majority), but included studies examining caregivers and healthcare providers as well. Studies examining palliative oncology in geriatric populations are underrepresented considering the incidence and mortality rates due to cancer are highest in patients 65 years and older. [13] In 2006–2016, hospitals and countries became study populations, representing a shift in scope from patient-level to a more macroscopic, population based analysis. Very few randomized controlled trial (RCT) were used until 2006–2016. This increase may be a response to the paucity of high-quality clinical evidence due to the ethical and practical challenges of conducting RCTs in palliative care. [14, 15] Analysis of the literature reveals that palliative oncology studies consistently rely on interviews and surveys, which have the potential of introducing greater subjectivity into the data.

An ongoing challenge of this relatively young field is the concentration of highly-cited literature coming from a select number of countries. Within these countries, such as the United States, studies are originating most often from handful of institutions. This is in contrast to research in other fields of oncology, particularly in drug development or therapeutic cancer clinical trials, which have robust involvement from Europe and East Asia. While the United States led in absolute terms of publications, Switzerland, Canada, and Sweden led as a factor of their total population in 2005 during the 1995–2005 period. During 2006–2016, Canada again remained a relatively productive source of published studies for its 2015 population size along with the Netherlands and Israel. This may reflect differences on how healthcare systems or social factors influence the recognition of palliative care as a distinct specialty. Regardless, our analysis shows an influx of new investigators making significant contributions to the field since 2006. Although no author remained amongst the 5 most published between the two time frames, the top authors in 2006–2016 produced over half (*n* = 54) of the studies as compared to only one-third (*n* = 34) of the top authors in 1995–2005. Focused but high-impact journals such as *Journal of Clinical Oncology* are the preferred outlet for publishing their work. Since research publication is the currency of career advancement in academia and foundation for improvement in clinical practice, these investigators and journals could offer valuable resources for future collaboration and publication.

The inherent bias of this analysis is that it favours older articles and may neglect recently published studies that, while significant, may have less citations. [16] In an attempt to control for lead-time publication date, the studies have been ranked based on number of citations per year, which will limit the effect of the absolute number of citations. However, more recently published articles may still not have had adequate time for inclusion in the top 100. Recently published articles with sufficient citations may have added importance, particularly those in Table 3. Although citation analysis is a frequently employed tool in scientific literature evaluation, number of citations may not necessarily correlate with a study’s impact and is an imperfect proxy for determining significance. [17] This analysis is unable to control for disproportionate citation due to institutional, language, or self-citation biases. Of note, several studies included subjects with non-malignant disease, resulting in a mixed study population. This may limit the ability to directly infer all findings to an exclusive cancer population.

By examining the 100 most-cited palliative oncology studies in 1995–2005 and 2006–2016, we were able to identify and analyze research trends across the literature. Existing palliative care studies in oncology largely focused on multiple–as opposed to single–cancer types, addressed the impact of palliative care on communication and end-of-life decisions, and were rarely RCTs. Early questions of pain, symptoms, and survival in palliative oncology have been replaced with studies addressing access to care and healthcare utilization. The relation of religion and spirituality to the field also has recently become a frequent area of investigation. Such information may prove useful in directing future study and highlighting the ongoing need for high-quality clinically-based research.

## Conclusions

Palliative oncology care is increasingly being delivered as a distinct service, ushering in a new era of research questions. A unique billing code for palliative care will allow investigators to study questions related to adoption of palliative care and its effect on healthcare utilization/cost of care using electronic medical record data and large datasets of medical claims. Oncology practices are now integrating patient reported outcomes into routine clinical care. This will provide a rich source of relevant, patient centric data where palliative oncology-specific quality metrics can be developed and tailored to individual cancer type and clinical setting (e.g. urban vs. rural, academic vs. community). Best illustrated by the seminal paper by J.S. Temel et al. (2010), more insight about the application of palliative oncology can be obtained from disease-specific randomized control trials. Ideally such rigorous research will propagate beyond a select number of well represented institutions and countries to incorporate the global medical community.

## Additional files

Additional file 1: **Table S1.** Top 100 most-cited palliative oncology studies in 1995-2005. (XLSX 21 kb)

Additional file 2: **Table S2.** Top 100 most-cited palliative oncology studies in 2006-2016. (XLSX 23 kb)

## References

[1] Clark D (2007). From margins to Centre: a review of the history of palliative care in cancer. The Lancet Oncology.

[2] Loscalzo MJ (2008). Palliative care: an historical perspective. Hematol Am Soc Hematol Educ Program.

[3] Payne SA, Turner JM (2008). Research methodologies in palliative care: a bibliometric analysis. Palliat Med.

[4] Tieman JJ, Abernethy A, Currow DC (2010). Not published, not indexed: issues in generating and finding hospice and palliative care literature. J Palliat Med.

[5] Sepulveda C, Marlin A, Yoshida T, Ullrich A (2002). Palliative Care the World Health Organization’s global perspective. J Pain Symptom Manage.

[6] Mehta NJ, Khan IA, Mehta RN, Tejani F, Vasavada BC, Sacchi TJ (2001). End-of-life care-related publications in cardiology journals. Am J Cardiol.

[7] Hui D, Parsons HA, Damani S, Fulton S, Liu J, Evans A, De La Cruz M, Bruera E (2011). Quantity, design, and scope of the palliative oncology literature. Oncologist.

[8] Sladek R, Tieman J, Fazekas BS, Abernethy AP, Currow DC (2006). Development of a subject search filter to find information relevant to palliative care in the general medical literature. J Med Libr Assoc.

[9] United Nations DoEaSA (2004). Population Division: World Population Prospects: The 2004 Revision.

[10] United Nations DoEaSA (2015). Population Division: World Population Prospects: The 2015 Revision.

[11] Ferlay J, Soerjomataram I, Dikshit R, Eser S, Mathers C, Rebelo M, Parkin DM, Forman D, Bray F (2015). Cancer incidence and mortality worldwide: sources, methods and major patterns in GLOBOCAN 2012. Int J Cancer.

[12] Services CfMM (2015). CMS Finalizes 2016 Medicare Payment Rules for Physicians, Hospitals & Other Providers.

[13] Yancik R (1997). Cancer burden in the aged: an epidemiologic and demographic overview. Cancer.

[14] Grande GE, Todd CJ (2000). Why are trials in palliative care so difficult?. Palliat Med.

[15] Tieman J, Sladek R, Currow D (2008). Changes in the quantity and level of evidence of palliative and hospice care literature: the last century. J Clin Oncol.

[16] Seglen PO (1998). Citation rates and journal impact factors are not suitable for evaluation of research. Acta Orthop Scand.

[17] Garfield E (1972). Citation analysis as a tool in journal evaluation. Science.

